# The dilemma of trade samples and the importance of museum vouchers—caveats from a study on the extinction of Steller's sea cow: a comment on Crerar *et al.* (2014)

**DOI:** 10.1098/rsbl.2015.0149

**Published:** 2016-02

**Authors:** Nicholas D. Pyenson, James F. Parham, Jorge Velez-Juarbe

**Affiliations:** 1 Department of Paleobiology, National Museum of Natural History, Smithsonian Institution, PO Box 37012, Washington, DC 20013-7013, USA; 2 John D. Cooper Archaeology and Paleontology Center, Department of Geological Sciences, California State University, Fullerton, CA 92834, USA; 3 Department of Mammalogy, Natural History Museum of Los Angeles County, Los Angeles, CA 90007, USA

## 1. Introduction

Crerar *et al.* [1] recently argued that a population of Steller's sea cows (*Hydrodamalis gigas*) persisted on St Lawrence Island (SLI), Alaska, well into historical time. If true, then Steller's sea cows were hunted to extinction in multiple places at different times in the past millennium [2]. We wish to highlight several serious issues with the data and practices that Crerar *et al.* [1] used to support their findings. Specifically, these concerns focus on the lack of voucher specimens and the source material (trade specimens) because they depart from accepted practices in natural history research.

## 2. The need to deposit specimens as museum vouchers

Crerar *et al.* [1] reported samples from SLI, listed in their tables 1 and 2 under the name ‘CRERAR’. For comparison, the authors used specimens accessioned at known repositories (i.e. the University of California Museum of Paleontology, Berkeley, CA, USA; and the Smithsonian Institution's National Museum of Natural History, in Washington, DC, USA) for samples collected from Bering Island, Russia. Because it is unclear where the SLI samples are currently located, they are not directly traceable and nor are they, by implication, openly accessible for research. Moreover, the authors did not provide any images of the bone samples attributed to *H. gigas*. For the initial identification of the rib bone fragments, the authors point to comparisons with more complete material at the Smithsonian Institution, and credit C. W. Potter, the collections manager, with confirming the identity. While fragmentary material is difficult to identify, the authors' line of reasoning is an argument made from authority, rather than demonstrated by evidence. The authors could have relied on diagnosable morphological traits, for example via histological section [3]. Although Crerar *et al.* [1] confirmed their initial identification with DNA, the presentation of rare specimens demands standard documentation (e.g. images, lists of diagnostic characters, detailed geographical and stratigraphic data). Without multiple lines of evidence to substantiate vouchers, and their attendant data, claims about sample identity and provenance cannot be verified. Although the samples could be eventually deposited into a museum collection, such subsequent action undermines any downstream voucher tracking that should have been accomplished at the time of publication.

## 3. The dilemma of purchased specimens from extinct taxa

The samples that Crerar *et al.* [1] reported from SLI were obtained privately, from material destined for the trade market. Crerar *et al.* [1] provide extensive second-hand testimony with the traders who collected the material, mentioning documentation such as airline receipts, to assert their provenance. Crerar *et al.* [1] used assurances as a form of justification, but this line of reasoning is not verifiable. A similar situation, with a far different outcome, occurred recently with the report of new skeletal material from *Spinosaurus*, a large Cretaceous theropod dinosaur from North Africa. Ibrahim *et al.* [4] initially noted fragmentary material from a local collector in Morocco, where abundant fossils are collected for trade markets. Subsequent work in museum collections by Ibrahim *et al.* [4] led to the relocation of the original collector, who directed them to the *Spinosaurus*-bearing locality. In this case, serendipity and diligent detective work resulted in clear geographical and stratigraphic provenance, and a wealth of additional material. By contrast, Crerar *et al.* [1] did not trace the origin of the material to a verifiable site; instead, they presented tantalizing accounts that beg more detailed investigation. More crucially, it is not clear how Crerar *et al.* [1] acquired the material from SLI for their study. Museum vouchers are usually donated in a legal framework and, in some cases, institutions with access to appropriately designated funding can purchase rare specimens. However, the high dollar values of vertebrate fossils underscore the ethical quandaries that accompany any purchases of such material. Some professional scientific societies (e.g. the Society of Vertebrate Paleontology) have passed explicit by-laws to guide members about the ethics of purchasing fossils, largely in step with recent legislation in the USA (i.e. the Paleontological Resources Preservation Act) to protect fossils collected on US federal lands.

We wish to emphasize that the discovery of historical Steller's sea cow material from SLI, about 1500 km away from the only other known historical remains of this extinct species [2], is important. Crerar *et al.* [1] provided valuable isotopic and radiocarbon data in their analyses, accessioned DNA sequence data in standard repositories, and raised intriguing questions about the pattern of historical extinction for this enigmatic marine mammal species. However, purchasing specimens of uncertain provenance from commercial collectors is not the only pathway for recovering natural history data from remote and difficult to access locations. For example, it is entirely possible that *H. gigas* specimens are vouchered, either unidentified or misidentified, in existing collections from SLI midden sites with multiple layers [5]. Vouchers in natural history museum collections have scientific value only with coordinating, attendant data that pin them in historical time, place and Earth history. Such parameters are essential for records of rare or extinct taxa. By using uncatalogued material with uncertain provenance, Crerar *et al.* [1] provided insufficient evidence for their conclusions, creating legacy problems for downstream research on their findings. We encourage the authors to deposit these important specimens into a natural history collection as soon as possible, and hope that future work will better establish the presence of *H. gigas* on SLI.
